# Plastic Deformation Modes of CuZr/Cu Multilayers

**DOI:** 10.1038/srep23306

**Published:** 2016-03-17

**Authors:** Yan Cui, Oscar Torrents Abad, Fei Wang, Ping Huang, Tian-Jian Lu, Ke-Wei Xu, Jian Wang

**Affiliations:** 1 Statekey Laboratory for Mechanical Behavior of Material, Xi’an Jiaotong University, Xi’an, 710049, People’s Republic of China; 2 INM-Leibniz Institute for New Materials, Campus D2 2, 66123 Saarbruecken, Germany; 3 State Key Laboratory for Mechanical Structure Strength and Vibration, Xi’an Jiaotong University, Xi’an, 710049, People’s Republic of China; 4 Mechanical and Materials Engineering, University of Nebraska-Lincoln, Lincoln, NE 68588 USA

## Abstract

We synthesized CuZr/Cu multilayers and performed nanoindentation testing to explore the dependence of plastic deformation modes on the thickness of CuZr layers. The Cu layers were 18 nm thick and the CuZr layers varied in thickness from 4 nm to 100 nm. We observed continuous plastic co-deformation in the 4 nm and 10 nm CuZr − 18 nm Cu multilayers and plastic-induced shear instability in thick CuZr layers (>20 nm). The plastic co-deformation is ascribed to the nucleation and interaction of shear transformation zones in CuZr layers at the adjacent interfaces, while the shear instability is associated with the nucleation and propagation of shear bands in CuZr layers. Shear bands are initialized in the CuZr layers due to the accumulated glide dislocations along CuZr-Cu interfaces, and propagate into adjacent Cu layers via slips on {111} plane non-parallel to the interface. Due to crystallographic constraint of the Cu layers, shear bands are approximately parallel to {111} plane in the Cu layer.

Amorphous metallic alloys or metallic glasses (MGs) have attracted much attention due to their excellent physical, chemical, and mechanical properties^1^,^2^. However, their applications are still limited due to the propensity for catastrophic failure that is associated with formation and rapid extension of shear bands^2^. Various approaches have been developed to prolong the plasticity of metallic glasses through constraining/suppressing formation and extension of shear bands. For example, MGs composites exhibit an improved plasticity because phase boundaries smear or blunt the localized shear deformation of shear bands^3^,^4^,^5^,^6^,^7^. By selecting appropriate composition of MGs, the plasticity of MGs can also be improved through nucleating multiple fine shear bands during mechanical deformation in lieu of rapid growth of shear bands^8^. The geometrical design of MGs samples also shows the potential for changing mechanical properties. Under compression, deflected shear bands with a wavy shape are generated in MGs samples with small dimensions in the directions perpendicular to the applied load, suppressing rapid extension of shear bands^9^,^10^. These studies suggest that plasticity enhancement in MGs can be achieved by tuning MGs compositions or microstructures that tailor mechanical characteristics of formation, extension and growth of shear bands in MGs.

Metal/MGs layered composites have been fabricated^11^,^12^,^13^,^14^ because of the potential enhancement of mechanical properties (strength and ductility) like what has been observed in crystalline metal/metal and metal/ceramics nanolayered multilayers^15^,^16^,^17^,^18^,^19^,^20^,^21^,^22^,^23^,^24^,^25^. These studies suggest a transition of plastic deformation modes from pronounced shear banding to homogeneous deformation as layer thickness decreases. Thus, multilayers consisting of amorphous and crystalline phases may constrain the formation and propagation of shear bands and enable the plastic co-deformation between MGs and metallic layers as the thickness of MGs layers is carefully tuned.

We fabricated nanolayered CuZr/Cu multilayers with alternating layers of amorphous CuZr and crystalline Cu layers (referred to as CuZr/Cu multilayers). Cu layers had a thickness of 18 nm in all multilayers. CuZr layers had different thicknesses of 4, 10, 20, 40, 75, and 100 nm, respectively. Micromechanical tests were conducted under a Nanoindenter-XP with Continuous Stiffness Measurement (CSM) mode at room temperature^26^. The indentation depth reached the maximum of 800 nm with a constant strain rate of 0.1 s^−1^. The morphologies of the indented samples were characterized using scanning electron microscope (SEM). Using a Nanoindenter-XP system (MTS, Inc.), the hardness of the studied samples can be automatically recorded as a continuous function of surface penetration depth by CSM mode. The ultimate hardness was calculated by averaging the values of the hardness obtained within a certain depth range of 4 ~ 6 times bilayer thickness, which is smaller than 1/5 of the total thickness of the multilayers.

## Results

**Figure 1:**
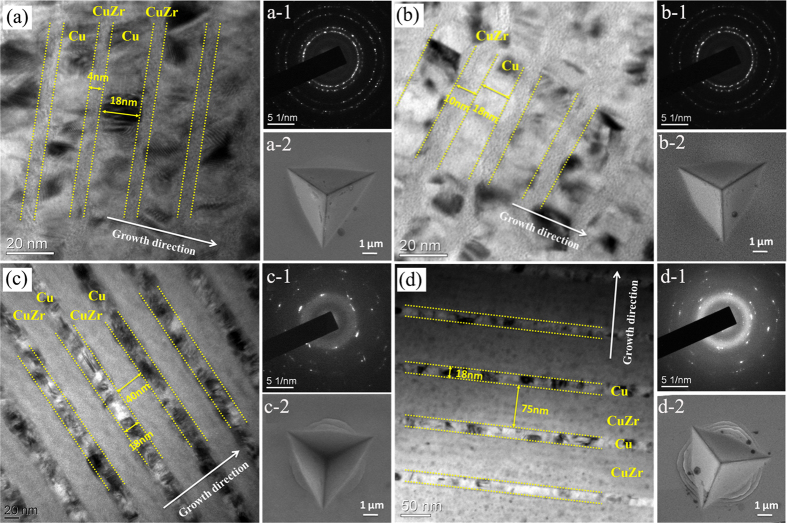
Microstructure of the selected CuZr/Cu multilayers before and after indentation testing. (**a**–**d**) the bright-field TEM images of cross-sectional CuZr/Cu multilayers with the amorphous layer thickness of 4 nm, 10 nm, 40 nm and 75 nm, respectively. (X-1) and (X-2) are the diffraction patterns and the SEM images of the respective multilayers. X represents (**a**–**d**).

Microstructural characterization of CuZr/Cu multilayers using scanning electron microscope (SEM), transmission electron microscope (TEM), and X-ray diffraction (XRD) reveals several features as shown in Fig. 1 and Figure S1 in the Supplementary: (i) CuZr/Cu composites have continuously layered structure; (ii) Cu grains are of single crystal across the layer thickness and the grain size is comparable to the layer thickness; and (iii) there is no apparent columnar structure. Thus, the CuZr/Cu multilayers exhibit a nanocrystalline, layered microstructure.

The cross-sectional TEM micrographs of the selected multilayers, 4 nm CuZr − 18 nm Cu, 10 nm CuZr − 18 nm Cu, 40 nm CuZr − 18 nm Cu, and 75 nm CuZr − 18 nm Cu, along with their corresponding diffraction patterns, are shown in Fig. 1, respectively. The diffraction patterns reveal a <111> fiber texture for the Cu layers. The distribution of grains size was determined from the TEM micrographs by measuring grain diameters in a fixed direction parallel to the interface (Figure S2 in the Supplementary). The average grain size in the Cu layers is estimated to be about 20 ~ 30 nm, comparable to the layer thickness.

The hardness of the CuZr/Cu multilayers are measured to be 4.82 GPa, 5.81 GPa, 6.32 GPa, 6.62 GPa, 7.25 GPa, and 7.06 GPa with a maximum error bar of 0.25 GPa for the CuZr layer thicknesses of 4 nm, 10 nm, 20 nm, 40 nm, and 100 nm, respectively. This shows an increasing trend with the increase of the CuZr layer thickness. SEM images of the selected multilayers after the indentation testing reveal the formation of shear bands as the CuZr layer thickness exceeds 20 nm while the homogenous plastic deformation in the 4 nm CuZr − 18 nm Cu and the 10 nm CuZr − 18 nm Cu multilayers.

**Figure 2:**
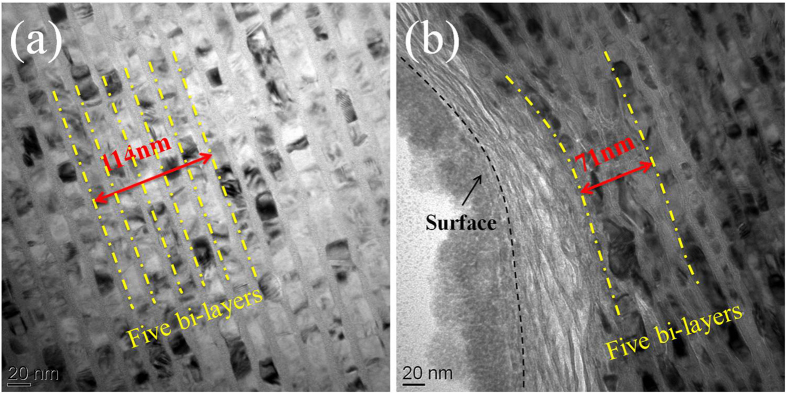
TEM images of the 4 nm CuZr/Cu multilayers (a) before and (b) after intentation testing. The first five bi-layers underneath the indenter experience a huge amount of deformation, corresponding to a compression strain of 38%.

The microstructure of the indented multilayers is characterized via TEM. Figure 2 shows the cross-sectional TEM images of the 4 nm CuZr − 18 nm Cu multilayer. Underneath the indenter, the 4 nm CuZr − 18 nm Cu multilayer experiences a huge amount of plastic deformation for both Cu and CuZr layers without the formation of shear bands or cracks in CuZr layers. In the first five bilayers underneath the indenter, the total thickness is reduced from 114 nm to 71 nm, corresponding to an average compression strain of 38%. In the next five bilayers (from the sixth to the tenth bilayers), the average compression strain is about 30%. This indicates that the Cu and CuZr layers in the 4 nm CuZr − 18 nm Cu multilayer plastically co-deform. We will discuss the co-deformation mechanism in the following sections.

**Figure 3:**
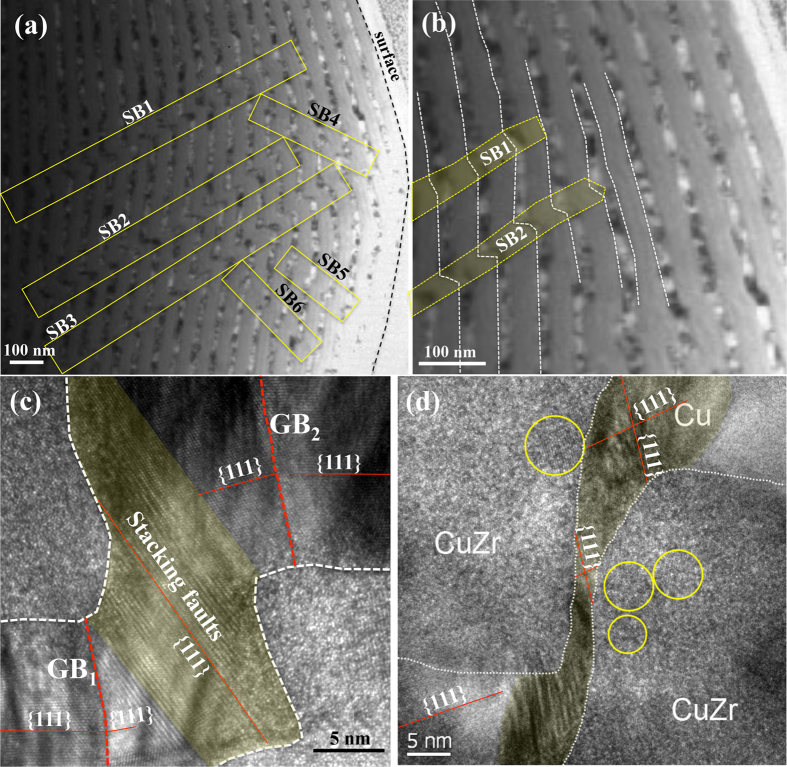
TEM images of the 40 nm CuZr/Cu multilayer after indentation testing. (**a**) Six shear bands outlined in the yellow rectangles and (**b**) the shear bands SB1 and SB2 end at the CuZr-Cu interfaces. (**c**,**d**) slip bands in the Cu layers that formed associated with shear on {111} planes non-parallel to the layers. The thin red line in (**c**,**d**) represents {111} planes in the Cu. Two tilt grain boundaries, GB_1_ and GB_2_, are marked in (**c**), indicating that shear also occurs on the {111} plane parallel to the layers. The regions outlined by the yellow circles show crystalline structure in the CuZr.

Differing from the 4 nm CuZr − 18 nm Cu multilayer, shear bands are formed in the 40 nm CuZr − 18 nm Cu and the 75 nm CuZr − 18 nm Cu multilayers. Figure 3 shows the cross-sectional TEM images of the 40 nm CuZr − 18 nm Cu multilayer. In Fig. 3a, we marked six shear bands in the rectangles, SB1 to SB6. Four intriguing features are characterized. 1) Shear bands did not nucleate from surfaces of the samples; 2) Shear bands continuously to cross through several crystalline and amorphous layers and end at the CuZr-Cu interfaces; 3) localized shear in the Cu layers is accomplished via slips on {111} planes; And 4) shear bands are approximately parallel to the {111} plane in the Cu layers (nonparallel to interface plane). The similar feature is also observed in the 75 nm CuZr − 18 nm Cu multilayers, as shown in Figure S3 in the Supplementary. In this scenario, slip bands are triggered in the Cu layers to accommodate the localized shear deformation associated with the extension of shear bands in the CuZr layers, as observed in Fig. 3c and schematically illustrated in Figure S4 in the Supplementary.

## Discussion

Our experiments so far revealed a profound size effect on plastic deformation of CuZr/Cu multilayers, i.e., CuZr/Cu multilayers plastically co-deform without the formation of shear bands when the CuZr layer thickness is less than 20 nm, but they deform associated with the formation and propagation of shear bands when the CuZr layer is thicker than 20 nm.

**Figure 4:**
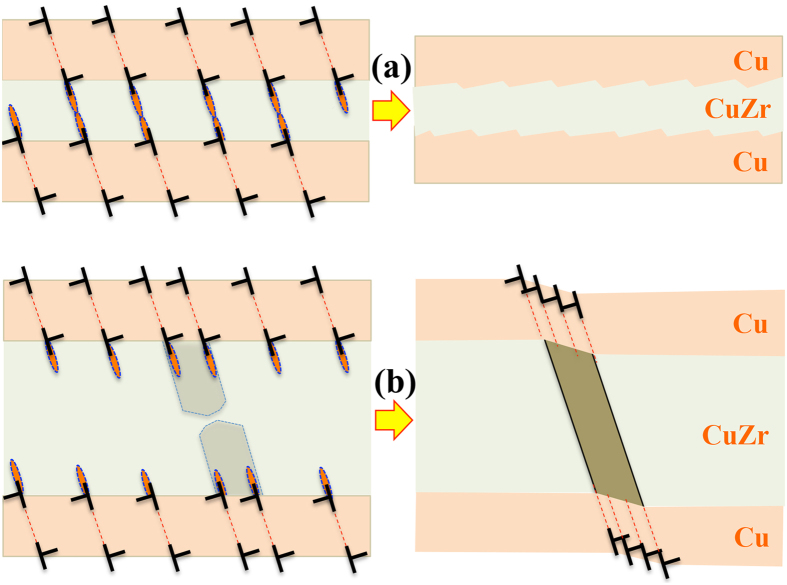
Schematics of plastic deformation modes with respect to the CuZr layer thickness. (**a**) Plastic co-deformation in the thin CuZr layers, showing plastic deformation in the Cu layers via slips on {111} planes and the formation and reaction of STZs in the CuZr layers. The red ellipses represent shear transformation zones (STZs). (**b**) The formation and propagation of shear bands in the thick CuZr layers, showing plastic deformation in the Cu layers via slips on {111} planes nonparallel to the layers and the formation of shear bands in the CuZr layers. The propagation of shear bands into the Cu layers is accomplished via the formation of slip bands on {111} planes. The shadow region in (**b**) indicates the coalescence of STZs along the interface, corresponding to the formation of shear bands. The dashed lines represent {111} planes nonparallel to the layers.

The lack of shear banding in the 4 nm and 10 nm CuZr/Cu multilayers could be understood according to plastic co-deformation mechanisms. Noted that there exists a critical length scale of 10 ~ 20 nm for amorphous CuZr alloys, below which amorphous CuZr layers can plastically deform via the so-called shear transformation zones (STZs)^11^,^27^. Particularly for the laminated microstructure, plastic co-deformation of CuZr/Cu multilayers can be rationalized as follows. Plastic deformation commences in Cu layers via the nucleation and propagation of glide dislocations on {111} planes in Cu. Under compression normal to the layers, glide dislocations propagate in the Cu layer and deposit at the Cu-CuZr interfaces, as demonstrated by molecular dynamics simulations (Figure S5 and Movie in the Supplementary). Associated with the nucleation and propagation of glide dislocations in Cu layers, plastic incompatibility is generated between the Cu and CuZr layers, resulting tension stresses in CuZr layers and compression stresses in Cu layers. The deposited dislocation arrays at interfaces also produce tensile stresses in the CuZr layers^28^. According to linear elastic mechanics and dislocation theory, the tensile stress in CuZr layers is proportional to the layers thickness ratio of *h*_Cu_/*h*_CuZr_ and the extent of the plastic incompatibility. A higher tension stress is generated for the thinner CuZr layers. In addition, the interaction between the deposited dislocations in the two adjacent interfaces contributes to the shear stress on the plane non-parallel to the CuZr layers. Thus the shear stress on a plane non-parallel to the CuZr layer is composed of three components: the contribution from the tension stress due to the plastic incompatibility, the interaction force between the deposited dislocations in the adjacent interfaces, and the applied stress, as shown in Figure S6 in Supplementary. According to dislocation theory^29^, the interaction force increases with decreasing the CuZr layer thickness, and becomes a significant contributor when the CuZr layer thickness is <≈10b where b is the magnitude of the Burgers vector. Thus for the thinner CuZr layers, the shear stress on the plane non-parallel to the CuZr layers enables the formation of shear transformation zones (STZs) and the propagation through the layer, as illustrated in Fig. 4a. The deposited dislocations at interfaces act as stress/strain concentrators, facilitating the formation of STZs at interfaces in CuZr layers and achieving plastic deformation transmission from the Cu layer to the CuZr layer, as demonstrated by molecular dynamics simulations in Supplementary (Figure S7). The 4 nm CuZr − 18 nm Cu multilayer thus plastically co-deform between the CuZr and Cu layers in response to the mechanical compression.

With the increase of the CuZr layer thickness, the tension stress in the CuZr layers and the interaction force between the deposited dislocations at the adjacent interfaces decrease. Even though STZs are formed at interfaces, the propagation of the STZs across the entire CuZr layer is slowed down due to the reduced shear stress on the plane non-parallel to the CuZr layers. When increasing the applied compression stress, the resultant shear stress on the plane nonparallel to the CuZr layers eventually triggers the formation of shear bands by gathering STZs in the CuZr layers. The formed shear bands then propagate towards the adjacent interfaces, and consequently trigger plastic deformation in the Cu layers. Corresponding to the crystallography of the Cu layer, slips on {111} planes via either Shockley partial dislocations or full dislocations accommodate the localized shear deformation in the adjacent CuZr layers. As a consequence, slip bands are formed in the Cu layers parallel to {111} planes, constraining shear bands in the CuZr layers that are parallel to the {111} plane in the Cu layers. Accompanying the propagation of such shear bands, the shear stress in the front of the shear bands decreases, associated with the development of plastic deformation, and the shear bands are more likely to eventually stop propagation at the CuZr-Cu interfaces.

The formation of shear bands in MGs has been discussed according to two types of models^30^. The percolation of homogeneously activated STZs and the heterogeneous nucleation of SBs from extrinsic flaws such as casting defects or surface notches. Corresponding to plastic deformation mechanisms in layered composites, the accumulation and deposition of glide dislocations at the CuZr-Cu interfaces from the Cu layers provide a plenty of sources for nucleating either STZs or shear bands in the CuZr layers. For thin CuZr layers, STZs that are nucleated at the adjacent interfaces can directly react to each other, facilitating slip transmission in the CuZr layer. When increasing the CuZr layer thickness, the direct reaction of the STZs at the adjacent interfaces through the thickness is lack, instead that STZs along the interface gather, favoring the formation of shear bands. Thus, shear banding in CuZr layers became the major plastic deformation mode to accommodate plastic deformation in the adjacent Cu layers, achieving continuous plastic deformation in the CuZr/Cu multilayers.

Figure 4b illustrates the formation and propagation of shear bands in the thick CuZr/Cu multilayers. Corresponding to the accumulation and deposition of glide dislocations at the interfaces, STZs are non-uniformly formed along the interfaces. Under continuously loading with mechanical perturbation or thermal fluctuation, the coalescence of the neighboring STZs takes place and result in the formation of shear bands. The formed shear bands propagate towards the adjacent interface and result in plastic deformation via slips on {111} planes in the Cu layers. Corresponding to the localized shear deformation, slip bands are formed in the Cu layers and further trigger shear bands in the CuZr layers. Due to the development of plastic deformation in the CuZr/Cu multilayers, the stress drops and the propagation of shear bands stops. Since the slip in Cu layers mainly takes place on the {111} plane non-parallel to the interface, the propagation of shear bands is most likely parallel to the {111} plane. In addition, thickening shear bands is confined because the continuous propagation of shear bands along the shear direction is related to the formation and propagation of slip bands in the Cu layers. The thickening of slip bands in the Cu layer is constrained by a back stress that increases with thickening slip bands due to the geometrical and elastic constraint of the adjacent CuZr layers. This suggests that the catastrophic failure that usually occurs in amorphous materials could be suppressed corresponding to the formation of multiple fine shear bands inside Amorphous/Crystalline multilayers.

In summary, we studied mechanical response of CuZr/Cu multilayers by conducting nanoindentation testing. The experimental results presented here revealed that plastic co-deformation takes place in the fine CuZr/Cu multilayers and shear banding occurs in the thick CuZr/Cu multilayers. More importantly, shear bands are initiated in the CuZr layers from interfaces due to the accumulation of glide dislocations in Cu layers at CuZr-Cu interfaces, propagate across multiple CuZr and Cu layers, and most likely end at the CuZr-Cu interfaces. The localized shear deformation in the Cu layers is mainly carried over by slips on {111} planes, thus keeping the shear band parallel to the {111} plane in the Cu layers. The thickening of shear bands is also constrained because of the back-stress originating from the geometric and elastic constraint of the adjacent CuZr layers. Furthermore, the formation of multiple fine shear bands should prevail over the development of thick shear bands, enabling continuous plastic deformation and suppressing the catastrophic failure that usually occurs in amorphous materials.

## Methods

### Sample preparation

Nanoscale multilayers with alternating layers of amorphous CuZr metallic glass and NC Cu (referred to as C/A multilayers hereafter) were deposited at room temperature using the techniques of *radio frequency* and *direct current* magnetron sputtering, respectively. Si(100) substrate with a thermal oxide surface layer was employed, and the amorphous CuZr layer was composed of 55 at% Cu and 45 at% Zr.

### Mechanical testing and microstructure characterization

The microstructure of the CuZr/Cu multilayers was first examined by X-ray diffraction (XRD) using a horizontal General Electric *θ* − 2*θ* power diffractometer in continue-scanning mode with Cu-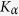 radiation, and subsequently by high-resolution transmission electron microscopy (HRTEM) using a JEOL JEM-2100F operating at 200 kV. Thin foils for TEM observation were prepared with twin-jet electro-polishing. Nanoindentation testing was carried out under a Nanoindenter-XP (MTS Corp., Oak Ridge, TN) with Continuous Stiffness Measurement (CSM) mode at room temperature. The indentations were performed at a maximum indentation depth of 800 nm and constant strain rate of 0.1 s^−1^.

## Additional Information

**How to cite this article**: Cui, Y. *et al.* Plastic Deformation Modes of CuZr/Cu Multilayers. *Sci. Rep.* **6**, 23306; doi: 10.1038/srep23306 (2016).

## Supplementary Material

Supplementary Information

Supplementary Movie
