# Supplementary material for: Plastic Deformation Modes of CuZr/Cu Multilayers
Source: Sci Rep. 2016 Mar 17;6:23306. doi: 10.1038/srep23306 (PMC4794738; doi:10.1038/srep23306)
Supplement: Supplementary Information [file srep23306-s1.pdf]

Supplementary

Plastic Deformation Modes of CuZr/Cu Multilayers

Yan Cui[1], Oscar Torrents Abad[2], Fei Wang[3,*], Ping Huang[1,*], Tian-Jian Lu[3], Ke-Wei Xu[1], Jian Wang[4,*]

[1]Statekey Laboratory for Mechanical Behavior of Material, Xi'an Jiaotong University, Xi'an, 710049, People's Republic of China
[2]INM-Leibniz Institute for New Materials, Campus D2 2, 66123 Saarbruecken, Germany
[3]State Key Laboratory for Mechanical Structure Strength and Vibration, Xi'an Jiaotong University, Xi'an, 710049, People's Republic of China
[4]Mechanical and Materials Engineering, University of Nebraska-Lincoln, Lincoln, NE 68588 USA

Correspondent authors: wangfei@mail.xjtu.edu.cn, huangping@mail.xjtu.edu.cn, jianwang@unl.edu.

Seven figures and one Movie are included as follows:

Figure S1. TEM images of 20 nm CuZr20 - 18 nm Cu and 100 nm CuZr – 18 nm Cu multilayers.

Figure S2. TEM images of 100 nm CuZr – 18 nm Cu and 4 nm CuZr – 18 nm Cu multilayers.

Figure S3.TEM images of the 75 nm CuZr/Cu multilayer after indentation testing.

Figure S4. Schematics of slip bands in the Cu layers, showing the shear on the {111}-planes.

Figure S5. Stress fields in 10 nm Al – 5 nm TiN in association with the deposited interface dislocations with the average spacing of 10 nm along the interfaces.

Figure 6. Atomic structures of 5 nm Cu - 2.5 nm CuZr multilayers in MD simulations.

Figure S7. Atomic structures around one CuZr layer in 5 nmCu - 2.5 nmCuZr multilayers.

Movie: Molecular dynamic simulation of 5 nm Cu - 2.5 nm CuZr multilayer.

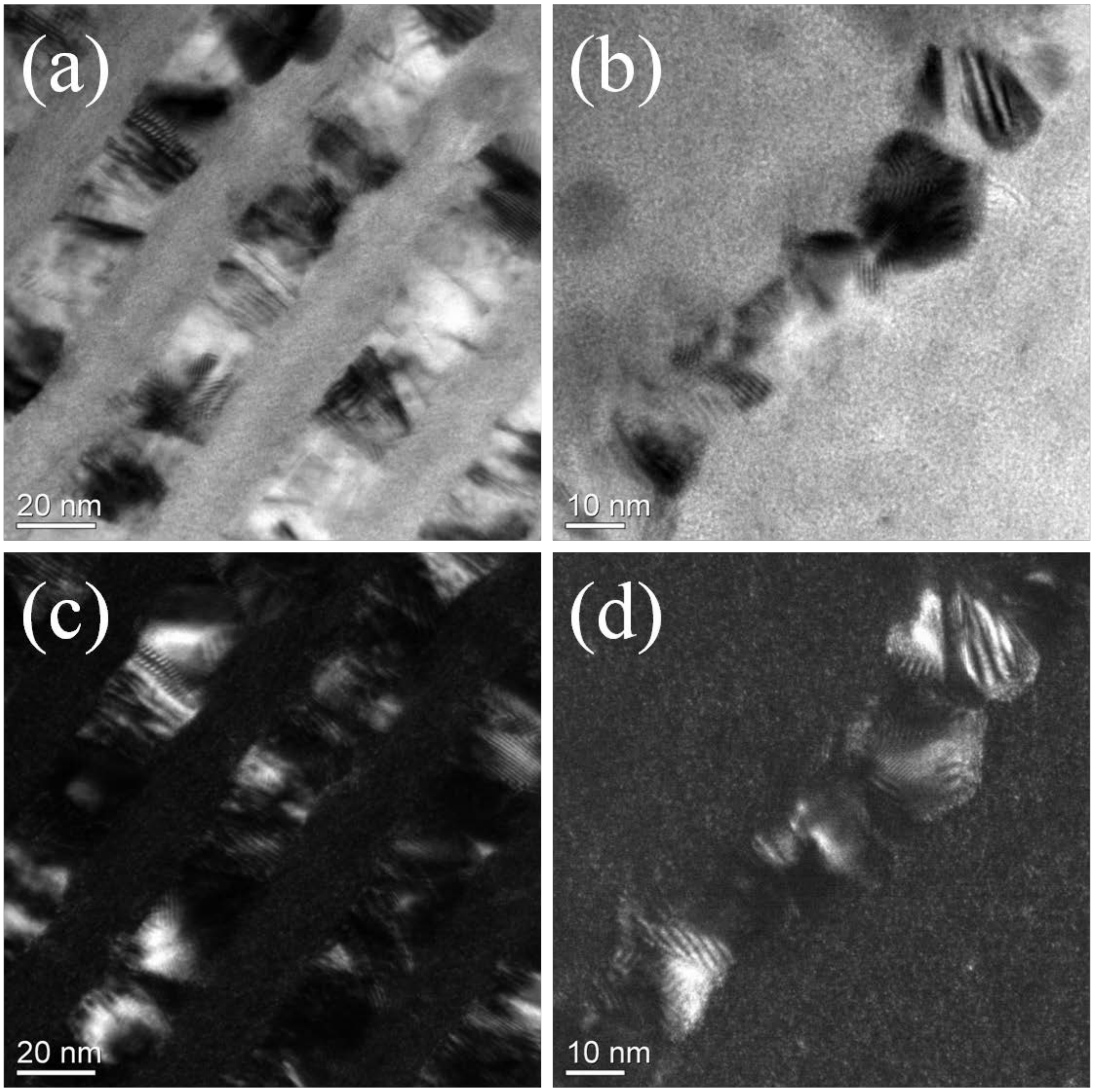

Figure S1. TEM images of (a) 20 nm CuZr20 - 18 nm Cu and (b) 100 nm CuZr – 18 nm Cu multilayers, (c) and (d) are the dark-field images of (a) and (b), respectively, which shows the continuous layered structure and no columnar structure.

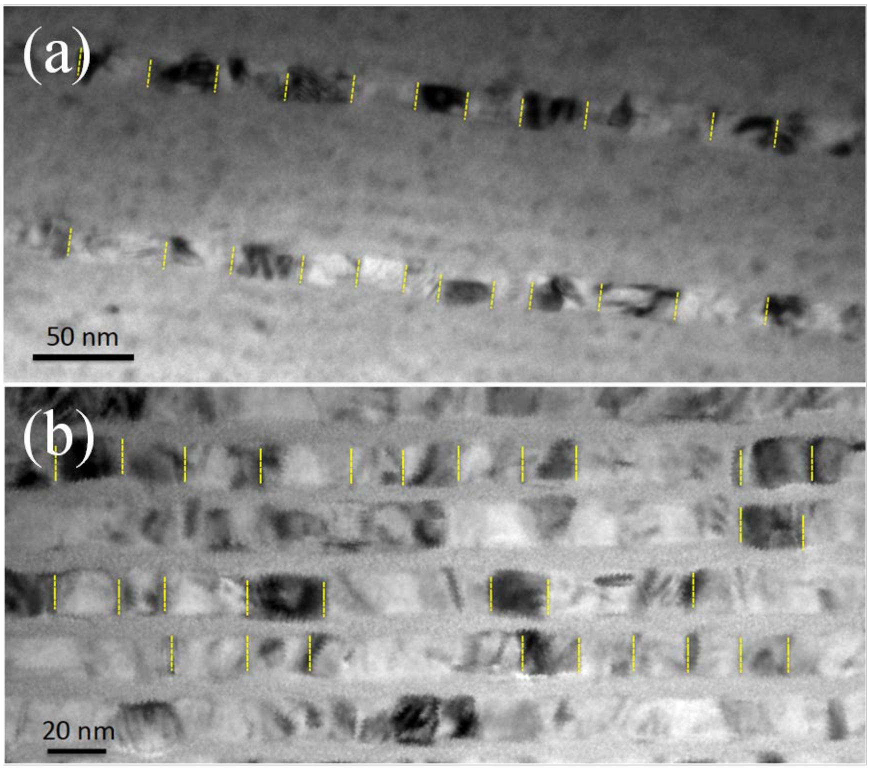

Figure S2. TEM images of (a) the 100 nm CuZr/Cu multilayers and (b) the 4 nm CuZr/Cu multilayers. The yellow dashed lines roughly indicate some of the grain boundaries in the Cu layers.

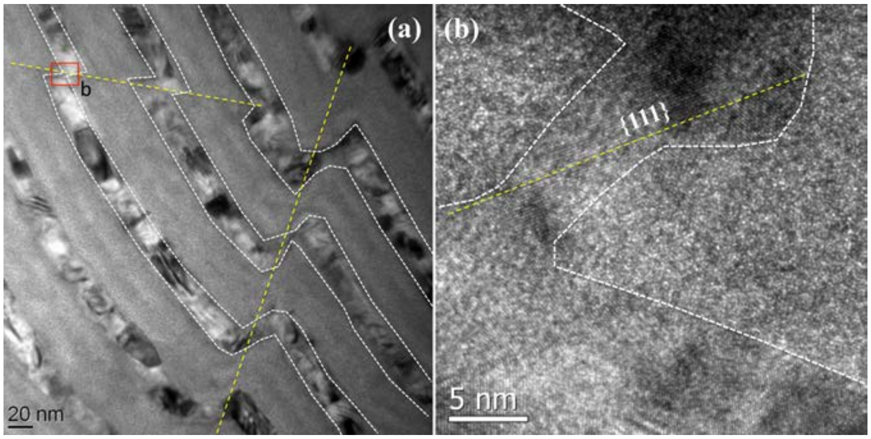

Figure S3.TEM images of the 75 nm CuZr/Cu multilayer after indentation testing. (a) Two shear bands are indicated along the yellow dashed shear planes and (b) the magnified TEM image of the red rectangle region in (a), showing the shear band is parallel to one {111} plane in the Cu. The yellow dashed lines in (a) indicate the shear plane. The right shear band has been discussed in Figure 3 in the main text and also is parallel to one {111} plane in the Cu.

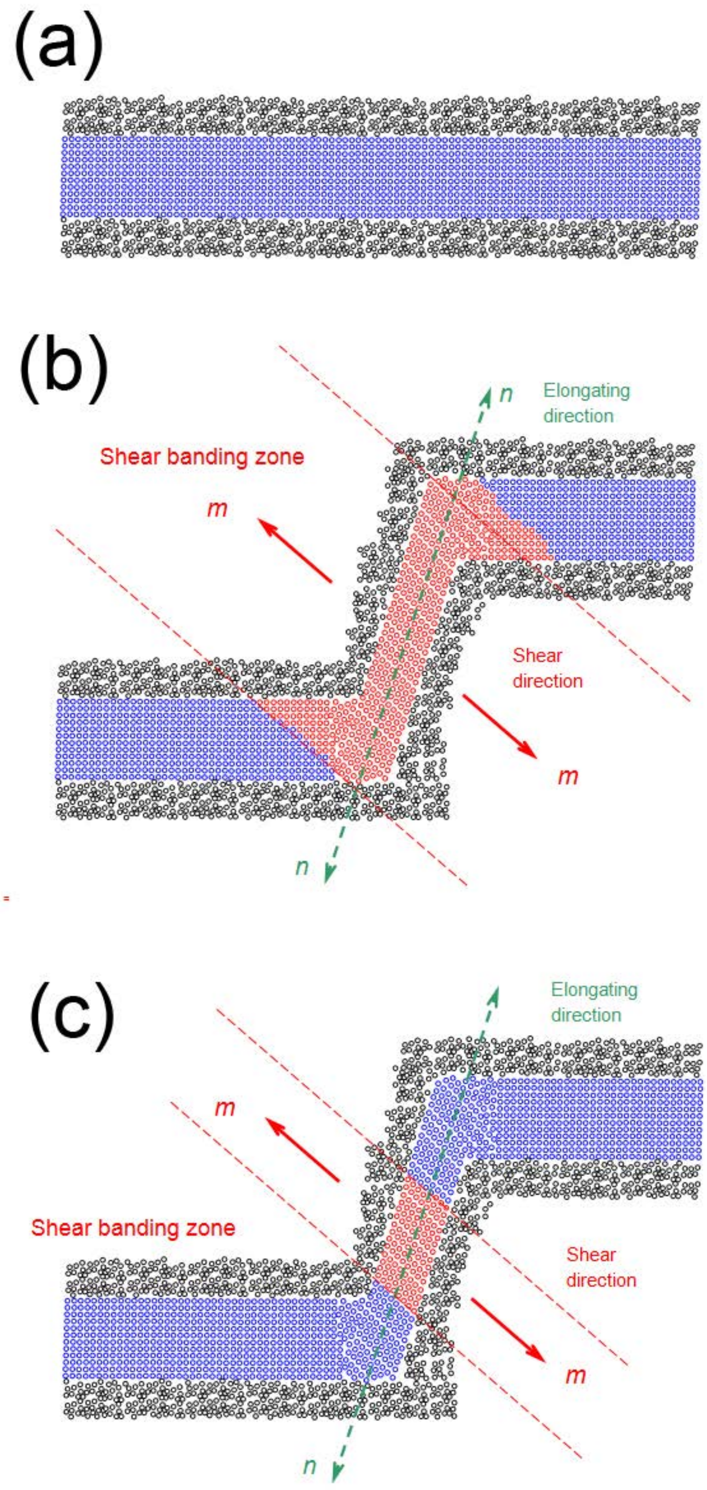

Figure S4. Schematics of slip bands in the Cu layers, showing the shear on the {111}-planes.

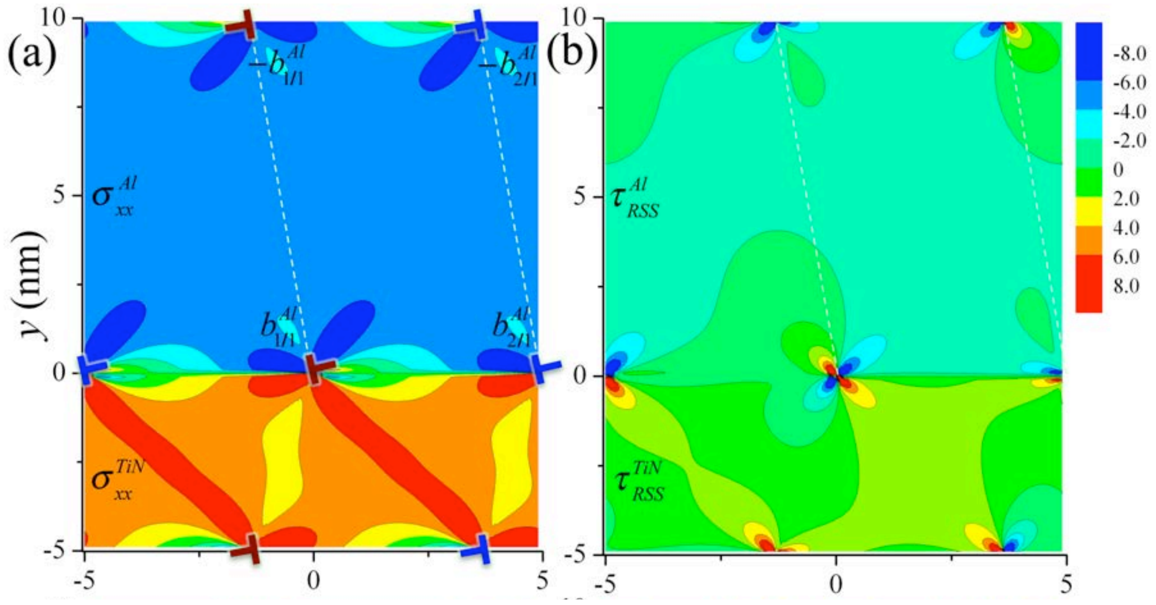

Figure S5. Stress fields in 10 nm Al – 5 nm TiN in association with the deposited interface dislocations with the average spacing of 10 nm along the interfaces (Wang, J. and Misra, A., Strain hardening in nanolayered thin films. *Curr. Opin. Solid State Mater.* **18** (1), 19-28 (2014).), showing (a) the normal stress parallel to the interface, compression in the Al layer and tension in the TiN layer, and (b) the resolved shear stress with respect to a glide plane (denoted as the dashed white lines), showing a locally high shear stress in the TiN layer due to the interaction of deposited dislocations on the adjacent interfaces.

Molecular dynamics simulations: We conducted molecular dynamic simulations of 5 nm Cu – 2.5 nm CuZr multilayers under uniaxial stress compression at temperature of 10 K with the compression strain rate of 10^8. The simulation cell contains 5 CuZr/Cu bilayers. The in-plane dimensions are 10.5 nm in the <112> direction in Cu and 11.5 nm in the <110> direction in Cu. Periodic boundary conditions are applied in all three directions. Dislocations are characterized using common neighbor analysis. We adopt the empirical interatomic potentials for Cu, Zr, and Cu-Zr developed by Cheng and Ma (Y. Q. Cheng and E. Ma, Atomic-level structure and structure–property relationship in metallic glasses. Prog. Mater. Sci. (2011).

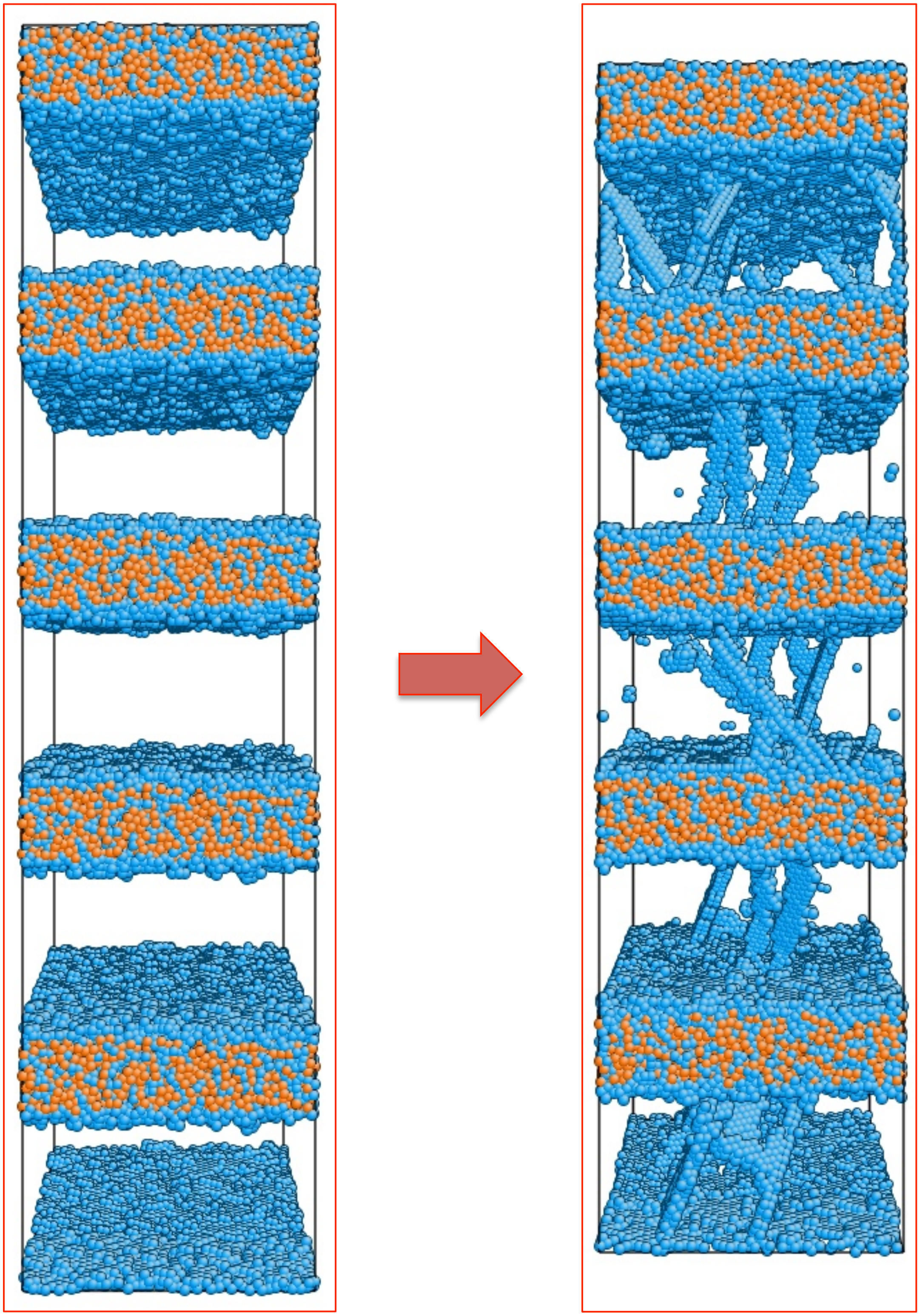

Figure 6. Atomic structures of 5 nm Cu - 2.5 nm CuZr multilayers, showing (a) initial structure and (b) the deformed structure containing glide dislocations in Cu layers under uniaxial compression at a strain of 6.5%. The details can be seen in Movie.

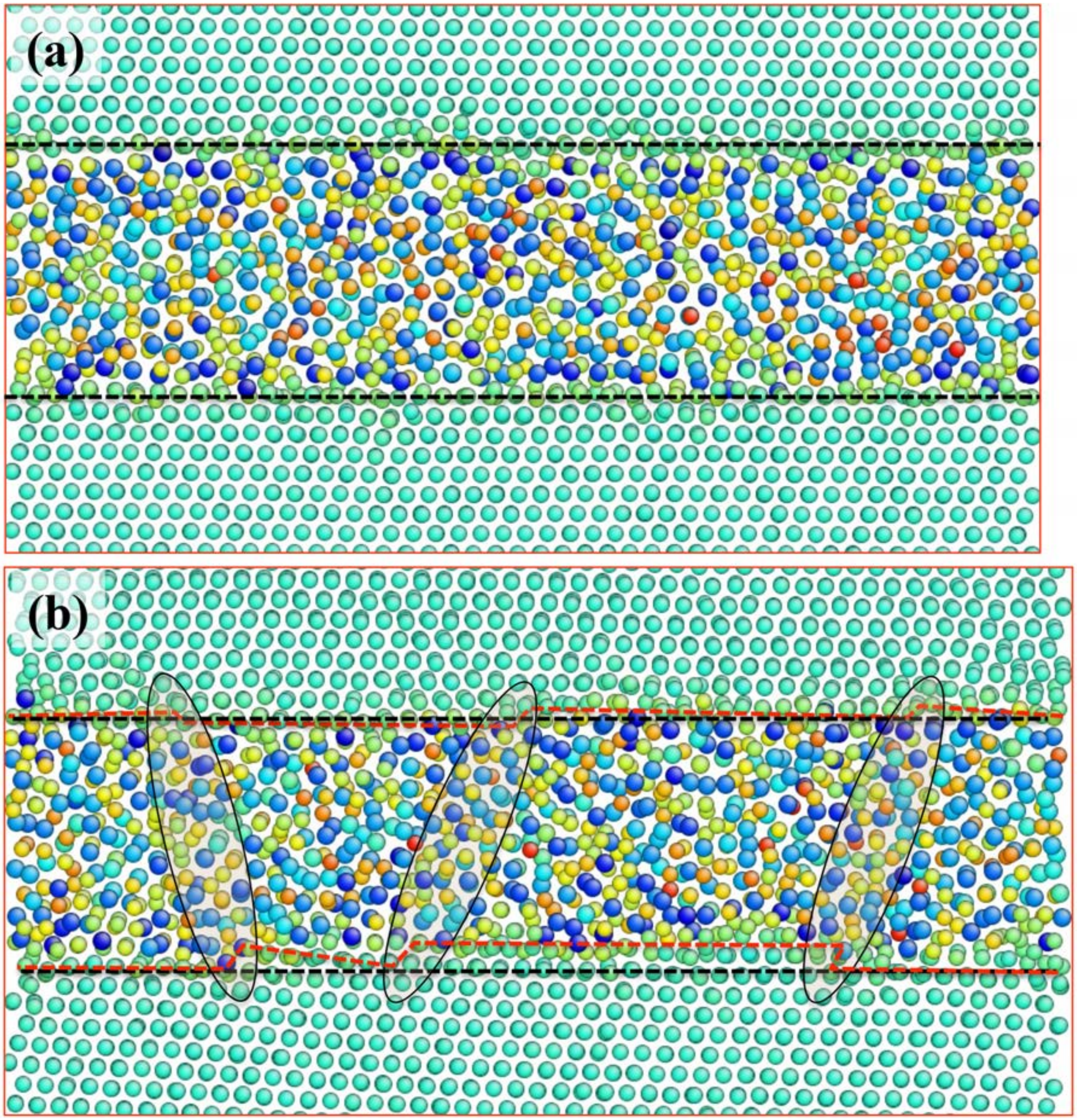

Figure S7. Atomic structures around one CuZr layer in 5 nm Cu - 2.5 nm CuZr multilayers, showing (a) initial structure and (b) the deformed structure under uniaxial compression at a strain of 8.0%. The black dotted lines indicate the original interfaces. The red dotted lines indicate the interfaces after 8% compression. The local shears across the CuZr layer are denoted by three ellipses.
